# *Alhagi maurorum* extract in combination with lytic phage cocktails: a promising therapeutic approach against biofilms of multi-drug resistant *P. mirabilis*

**DOI:** 10.3389/fphar.2024.1483055

**Published:** 2024-12-13

**Authors:** Arezoo Mirzaei, Bahram Nasr Esfahani, Mustafa Ghanadian, Jeroen Wagemans, Rob Lavigne, Sharareh Moghim

**Affiliations:** ^1^ Department of Bacteriology and Virology, School of Medicine, Isfahan University of Medical Sciences, Isfahan, Iran; ^2^ Department of Pharmacognosy, School of Pharmacy, Isfahan University of Medical Sciences, Isfahan, Iran; ^3^ Department of Biosystems, KU Leuven, Leuven, Belgium

**Keywords:** Alhagi maurorum, bacteriophage therapy, *Proteus mirabilis*, biofilm, combination therapy

## Abstract

Antimicrobial resistance (AMR) poses a significant global threat to public health systems, rendering antibiotics ineffective in treating infectious diseases. Combined use of bio compounds, including bacteriophages and plant extracts, is an attractive approach to controlling antibiotic resistance. In this study, the combination of phage cocktail (Isf-Pm1 and Isf-Pm2) and *Alhagi maurorum* crude extract (AME) was investigated in controlling biofilm-forming multi-drug resistant *P. mirabilis* isolates, *in vitro* and a phantom bladder model. The combination of AME and phage cocktails demonstrated no significant disparity in its ability to inhibit quorum sensing (QS) when compared to the individual control of AME alone. Following treatment with the combination of phage cocktail and AME at a 125 μg/mL concentration, the MDR *P. mirabilis* biofilm biomass was notably reduced by 73% compared to the control (P< 0.0001). The anti-biofilm effect was confirmed by Scanning Electron Microscopy (SEM). Moreover, in a bladder phantom model, there was a considerable decrease in encrustation levels compared to the control. The combined treatment resulted in a 1.85 logarithmic reduction in bacterial adhesion to Vero cells compared to the control. The real-time PCR results indicated significant downregulation of QS- and adhesion-related gens. The phage therapy, combined with AME, holds promising potential in reducing biofilm formation.

## Graphical Abstract

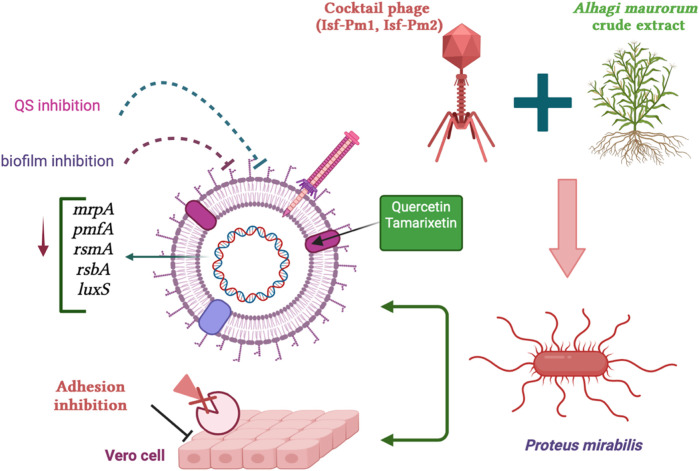

## Highlights

• Harnessing the Power of Nature: Bacteriophages and Plant Extracts Unite to Overcome Antibiotic Resistance
• Blasting Biofilms: Phage Cocktail and AME Reduce MDR *P. mirabilis* Biofilm Biomass by 73%
• Clearing the Path: Combination Therapy Significantly Reduces Encrustation Levels in a Bladder Phantom Model
• A Shield Against Adhesion: Phage Cocktail and AME Dramatically Reduce Bacterial Attachment to Vero Cells
• Silencing the Threat: Real-Time PCR Reveals Downregulation of QS- and Adhesion-Related Genes

## Introduction

Antimicrobial resistance (AMR) is recognized as a significant threat to public health systems worldwide. Infectious diseases can no longer be treated with antibiotics, presenting an uncertain future in healthcare (Chokshi et al., 2019). In recent years, the focus has shifted towards novel strategies to reduce antibiotic resistance levels, including combined therapies.

*Proteus mirabilis* is a significant pathogen in urinary tract infections (UTIs), particularly in complicated cases and catheter-associated urinary tract infections (CAUTIs). The incidence of *P. mirabilis* infections notably increases with prolonged catheterization, often affecting vulnerable populations such as patients in nursing facilities and long-term care institutions (Fox-Moon and Shirtliff, 2024).

These infections pose considerable clinical challenges due to the organism’s resistance to commonly used antibiotics. Furthermore, *P. mirabilis* can exacerbate complications such as urolithiasis, potentially leading to irreversible renal impairment. The progression of these infections can also result in serious conditions like bacteremia and sepsis, particularly as the bacteria utilize catheters as pathways for spreading through tissues. This resistance complicates treatment options and underscores the need for effective strategies to combat these infections (Chakkour et al., 2024).

Bacteriophages and plant extracts are being explored individually or in combination with conventional antibiotics to treat multidrug-resistant (MDR) infections (Cheesman et al., 2017). Bacteriophages are natural antibacterial agents capable of regulating bacterial populations by inducing bacterial lysis. The lytic activity of phages has been explored against gram-positive (Leprince and Mahillon, 2023) gram-negative bacteria (Shah et al., 2023), and MDR pathogens. Specific barriers to applying phages as routine therapy in humans include the emergence and evolution of phage resistance in bacteria and their narrow host range (Zalewska-Piątek, 2023). Combination of a phage cocktail with antibiotics and/or other antibacterial compounds, such as natural antimicrobial plant extracts, might reduce antibiotic consumption, control antibiotic resistance and phage therapy limitations (Pimchan et al., 2018). More investigation is needed to explore the possible synergetic effect of the combination of plant extracts and bacteriophages. It has been reported that *Alhagi maurorum* (*A. maurorum*), a traditional medicinal plant in the Middle East, is rich in phenolic and flavonoid compounds (Al-Jaber et al., 2011). These active ingredients have been shown various therapeutic benefits, including antiviral, antibacterial, anti-inflammatory, and anticarcinogenic activities (Ullah et al., 2020). The anti-biofilm activity of *A. maurorum* extract on *P. mirabilis* has been investigated previously (Mirzaei et al., 2022a). The virulent bacteriophages Isf-Pm1 and Isf-Pm2 have been isolated from municipal wastewater and were shown to be able to reduce the concentration of *P. mirabilis* from 10^9^ CFU/mL to 10^1.6 ^CFU/mL in 30 min when used as a cocktail at a multiplicity of infection (MOI) of 1 (Mirzaei et al., 2022b). In this study, we focused on the possible synergetic antimicrobial activity of *A. maurorum* extract (AME) in combination with the phage cocktail in an *in vitro* phantom biofilm model.

## Materials and methods

### *Alhagi maurorum* extracts (AME)

The crude extract of *A*. *maurorum* plant was prepared with hydroalcoholic method as described in our previous study (Mirzaei et al., 2022a). The extraction solvent utilized for the crude extract was water.

### Antibacterial activity of *Alhagi maurorum*

The minimum inhibitory concentration (MIC) of the AME was determined using the microbroth dilution method, following a previously described protocol (Veiga et al., 2019). In summary, stock solution of AME (2 mg/mL) was subjected to serial two-fold dilution in sterile U-bottom 96-well microplates containing Mueller Hinton Broth (MHB). Subsequently, 100 μL of MHB was added to all wells. A 100 μL of the extract (2 mg/mL) was poured into each well of the first row. Inoculum of 10 μL with a concentration of 10^8^ CFU/mL was added to each test well. The plates were then covered and incubated at 37°C for 24 h. Following incubation, the MIC was determined as the lowest concentration of the tested material that visibly inhibited bacterial growth. In the negative control well, no substances were added.

### Bacteriophages and bacterial isolates

Forty *P. mirabilis* isolates were collected from catheters at the intensive care units (ICUs) of reference hospitals in Isfahan, Iran, and identified by conventional biochemical method followed by PCR-sequencing of the *ureG* gene (Mirzaei et al., 2021). The local ethical committee of Isfahan University of Medical Sciences approved this study (IR.MUI.MED.REC.1401.405). Also, the participants provided written informed consent to participate in this study.

*P. mirabilis* ATCC7002 was used as a reference strain. The antimicrobial resistance pattern and biofilm formation of isolated bacteria was performed by disk diffusion and microtiter plate according to the Clinical and Laboratory Standards Institute (CLSI_2022_) guidelines and the O'Toole study (O’Toole, 2011). MDR isolates were classified as those resistant to at least one agent from three or more antimicrobial classes, according to Magiorakos et al. (2012). Twelve MDR and strong biofilm-forming clinical isolates were chosen for further investigation (Supplementary Table S1).

Pm Isf-1 and Pm Isf-2, lytic phages of *P. mirabilis* ATCC7002, were isolated from Municipal wastewater. The phage isolation and characterization are reported in our previous study (Mirzaei et al., 2022b).

### Phage-extract interaction assay

In order to test the direct effect of the extract on phage titer and plaque-forming ability, a coincubation assay was performed (Pimchan et al., 2018). Briefly, 1 mL of phage lysates (Pm Isf-1: 10^8^ PFU/mL, Pm Isf-2: 10^8^ PFU/mL) were sampled and supplemented with 1 mL of two-fold serial dilutions of plant extract (62.5–1,000 μg/mL). Phage lysate alone was used as positive control. The microtubes were incubated at 37°C for 24 h. The samples were centrifuged at 5,000 × g for 5 min, and the supernatants (phages) were titered by the double-layer agar method (Clokie et al., 2009).

### Quantitative anti-quorum sensing (anti-QS) assay

The anti-QS test was performed based on the ability to inhibit the production of the purple pigment violacein by *Janthinobacterium lividum (J. lividum)* ATCC 12472 according to Chenia’s method (Chenia, 2013).

### Anti-biofilm activity of phage-extract combination

To study the anti-biofilm activity of phage-extract on bacteria, a 96-well microplate dilution method was performed. A hundred microliter of the overnight culture of *P. mirabilis* (OD_620nm_ of 0.1) suspension were added to a 96-well polystyrene flat-bottomed plate and incubated at 37°C for 72 h. Then, the media was discarded, and the biofilms were washed with phosphate-buffered saline (PBS) (pH 7.2). Serial dilutions of AME (125–1,000 μg/mL) were prepared and were added to each biofilm-coated well in combination with the phage cocktail (1:1) at different MOIs (0.1, 1, 10). As a positive control, 100 μL of nutrient broth was added to the original biofilm of the *P. mirabilis* isolates. The treated plates were incubated for 18 h at 37°C. The biofilm biomass was stained by 0.1% crystal violet method and was quantified by measuring the OD_620_ using a microtiter plate reader (Infinite F50, Tecan) (Mirzaei et al., 2021). The experiment was conducted in triplicates.

### Adhesion assay

Vero cells (Pasteur Institute of Iran), seeded in 24-well plates (0.5 × 10^5^ cells/well), was overlaid with 200 µL of AME (125–1,000 μg/mL) and 200 µL of phage cocktail (MOIs 0.1–10). Then, 40 µL of the *P. mirabilis* suspensions (OD_620nm_ = 0.1) were added to the wells, followed by incubation at 37°C (5% CO_2_) for 3–6 h. The wells were washed three times with PBS to remove non-adherent bacteria. Cells were then treated with 400 μL of 0.025% Triton X-100 for 5 min at 37°C to detach and lyse the cell monolayer and suspended in 1.6 mL phosphate-buffered saline (PBS). Bacterial colonies were counted, and the number of bacterial colonies in the treated plates were compared to the control (Rocha et al., 2007).

### Biofilm inhibition of combined phage-extract in phantom bladder model

*In vitro* bladder models, as originally described by Stickler and Morgan (2006), were established and maintained at 37°C using a double-walled glass chamber. Briefly, after sterilization through of phantom bladder model, size 18 all-silicone Foley catheters were inserted, and retention balloons were inflated with 10 mL of sterile water. A sterile urine sample from a healthy male volunteer, free from urinary tract infections, was filtered and pumped into the bladder, creating a residual volume of 30 mL. *P. mirabilis* ATCC 7002 cell suspensions (10^8^ CFU/mL) were added to this residual urine to simulate late-stage infection and allowed to incubate for 1 h. After 45 min, the models received a single dose of a two-phage cocktail (2 × 10^10^ PFU/mL) and AME at the optimal concentration (125 μg/mL) in a volume of 1 mL, after which flow was restored. Viable cell counts in the residual bladder medium were measured at the beginning and end of the experiments, and pH levels were checked. Additionally, calcium deposition on the catheters was assessed using flame photometry 18 h after treatment. In this procedure, 1-cm catheter sections were placed in a solution of 95% ammonium oxalate and 5% oxalic acid, mixed vigorously for 3 min, and incubated at room temperature for 30 min. After removing the catheter sections, the solution was centrifuged at 3,000 × g for 10 min, and the supernatant was discarded. The remaining pellets were resuspended in 5 mL of 0.05 M perchloric acid, mixed well, and centrifuged again at 3,000 × g for 2 min to collect the supernatants. The calcium concentration in these supernatants was then measured using a flame photometer (Nzakizwanayo et al., 2016).

### Biofilm inhibition analysis using scanning electron microscopy (SEM)

The biofilm of *P. mirabilis* (10^6^ CFU/mL) was formed in a 6-well microtiter plate containing a glass coverslip and incubated at 37°C for 72 h. The coverslips were subsequently exposed to 1 mL of the respective phage cocktail (final 2 × 10^10^ PFU/mL of each phage) in combination with 1 mL of AME (optimal concentration 125 μg/mL) and incubated for 18 h. Then, the coverslips were rinsed two times with PBS and air-dried in an incubator set to 37°C for 20 h. The biofilms were fixed with 2.5% glutaraldehyde and dehydrated using a graded ethanol series (30%–100%) for 5 min. Subsequently, the biofilms were subjected to critical point drying, sputtered with gold, and observed under SEM (FEI, Tecnai G-2S Twin) using an operating voltage of 15 kV.

### Evaluation of QS and adhesion gene expression level by real-time PCR

Quantitative real-time PCR (qRT-PCR) assay was performed to determine the effect of the combination of phage - AME on the expression of QS and adhesion genes (*mrp*A, *pmf*A, *lux*S, *rsm*A, and *rsb*A) of *P. mirabilis*. Overnight pooled urine of *P. mirabilis* 7,002 was transferred to fresh urine, treated with an optimal concentration of AME (125 μg/mL) and phage cocktail (MOI 1:1), and incubated at 37°C for different time intervals (4, 16, and 48 h). Total RNA was extracted from bacterial cells using an RNA extraction kit (Jena Bioscience, Germany). The cDNA was synthesized according to the kit protocol (Jena Bioscience, Germany). qRT-PCR was performed on an ABI system (Applied Biosystems StepOne Plus™, United States). Each 20 µL reaction contained 2 × Master Mix (SYBR Green Ampliqon, Denmark), diluted cDNA (5 ng/L), primers (10 p.m. of each forward and reverse primer), and RNase-free ddH_2_O. The thermocycling conditions were as follows: 10 min, 95°C, followed by 40 cycles of 15 s at 95°C, 54 °C for 60 s 16 s rRNA was used as an internal control. The primers used in this study are mentioned in our previous study (Mirzaei et al., 2022b). All samples were run in triplicate. The conventional 2^−ΔΔCT^ method calculated the relative expression of target genes (Livak and Schmittgen, 2001).

### Determination of cell viability (MTT assay)

Vero cells (0.5 × 10^4^ cells/well) were seeded in 96-well microtiter plates in the presence of Dulbecco’s Modified Eagle Medium (DMEM, Gibco, United States) supplemented with 5% FBS (Gibco, United States) and incubated for 12 h in a humidified atmosphere with 5% CO_2_ at 37°C. A 100 μL of ten-fold serial dilutions of AME (62.5–1,000 μg/mL) and Phage (MOI_s_ 0.01–10) were added to each well. Cell viability was assessed 24 h post incubation by MTT assay, as previously described (Mirzaei et al., 2022b).

### Statistical analysis

All statistical analyses were carried out using GraphPad Prism9 (GraphPad Software, San Diego, CA, United States). All results were presented as mean and standard deviation (SD). A two-way ANOVA plus Dunnett’s multiple comparisons tests was used to analyze the phage statistically–extract coincubation assay results. P-values< 0.05 were considered statistically significant.

## Results

### Antibacterial activity of *A. maurorum* and the phage cocktail against *P. mirabilis* isolates

The antimicrobial activity of AME was previously assessed against strong biofilm-forming MDR *P. mirabilis* isolates. The results showed that the bulk plant extract had the best biofilm inhibition at a 125 μg/mL concentration against all isolate (Supplementary Figure S1) (Mirzaei et al., 2022a). A MIC assay on 12 selected isolates treated with AME demonstrated no antibacterial activity in any extract concentration. The lytic phages Isf-Pm1 (*Casjensviridae, Lavrentievavirus*, accession number OL741431) and Isf-Pm2 (*Straboviridae, Tequatrovirus*, accession number OL741432) have been previously shown to be active against the biofilm of the isolates efficiently. The optimal efficacy of phage cocktail was determined at MOI1 (Supplementary Figure S2) (Mirzaei et al., 2022b).

When the AME was tested for antiphage activity, no significant reduction of the phage count was observed at bulk concentrations of 1,000 mg/mL or lower (*p* > 0.05; Supplementary Figure S3).

### The anti-QS, antibiofilm, and adhesion activity of a combination of AME and phage cocktail

**FIGURE 1:**
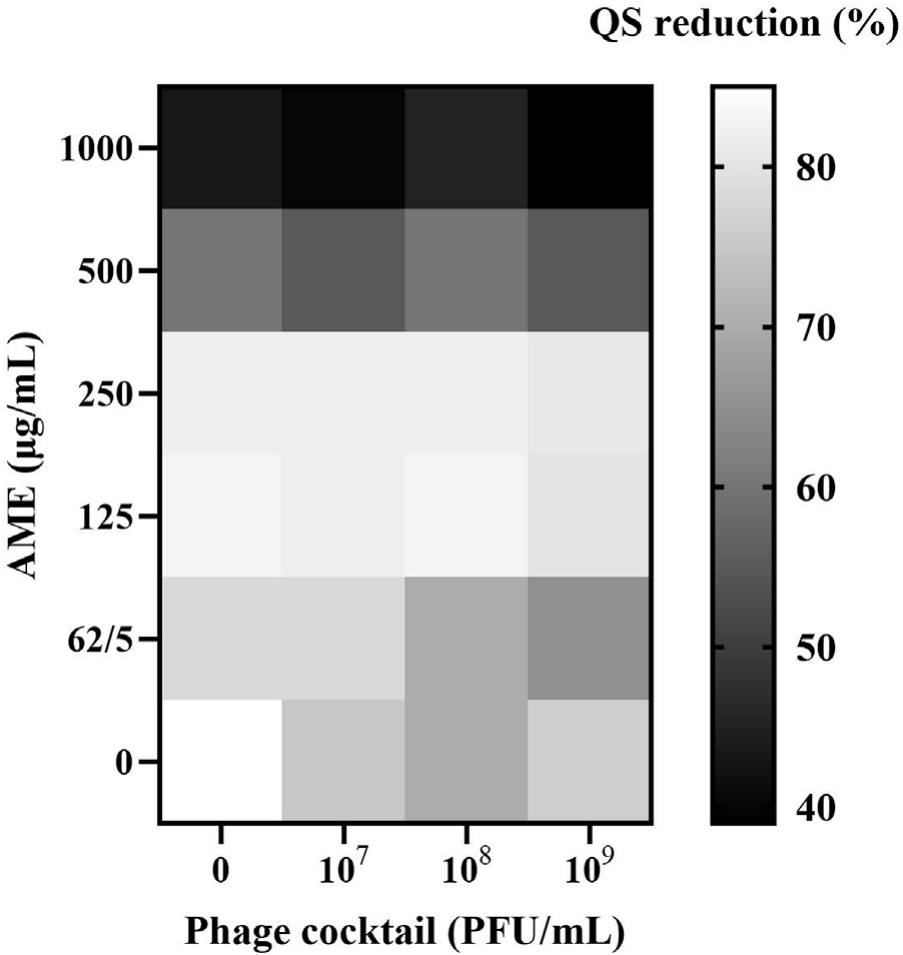
Effects of combined AME and Phage cocktail on QS activity using *J. lividum* ATCC 12472. Heatmap represents OD_620_ nm measurements of the violacein. Synogram represents the mean reduction (% of the control) of the treatment from the three replicates (P > 0.05). Eugenol was used as a positive anti-QS control, and DMSO was used as a negative control.

Lower concentrations of the plant extract (125–1,000 μg/mL) clearly caused an increase in the anti-QS activity of MDR isolates. Overall, the combination of AME and phage cocktails exhibited no significant difference in anti-QS activity when compared to AME individual control (*p* > 0.05, Figure 1). However, AME alone and in combination with the phage cocktail significantly reduced QS activity in all cases.

**FIGURE 2:**
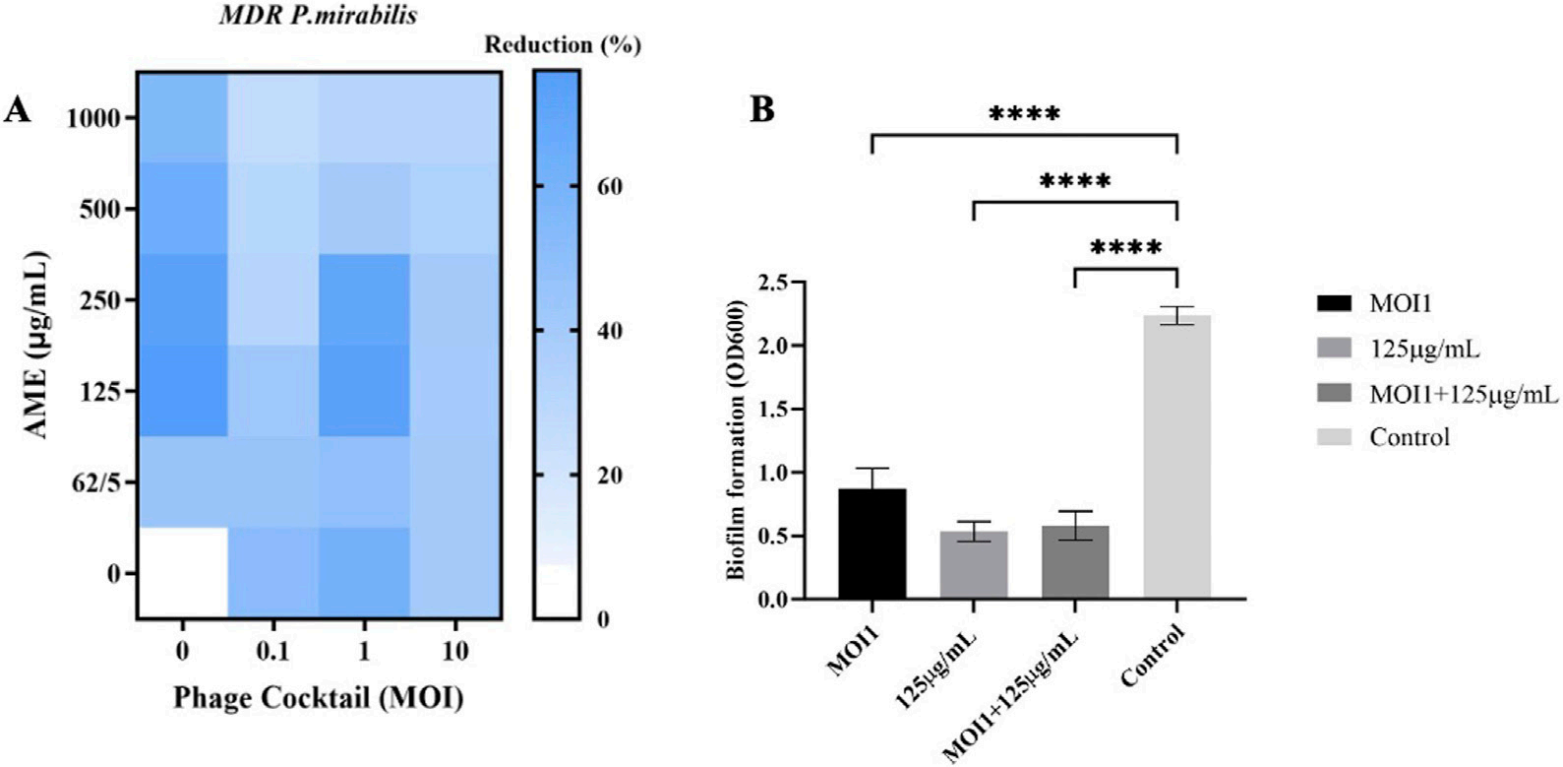
**(A)** Heat-map of combined phage cocktail-extract in biofilm of host bacteria. **(B)** Comparison of biofilm reduction percentage at different treatment with AME, Phage and combination. AME and Phage cocktail at different concentrations and MOIs. The statistical method used was the One-way ANOVA with Dunnett’s test.

The biomass of MDR *P. mirabilis* biofilm was significantly reduced by 73% after treatment with the combination of phage cocktail (MOI 1) and AME at 125 μg/mL compared to the control (*p* < 0.0001), and remained at a similar level for the extract-only therapy (Figure 2). Compared to combination therapy and control, the phage cocktail did not significantly alter bacterial multiplication at a high concentration (MOI 10). The combined treatment at higher concentrations did not show a significant effect on decreasing the bacterial biomass.

**FIGURE 3:**
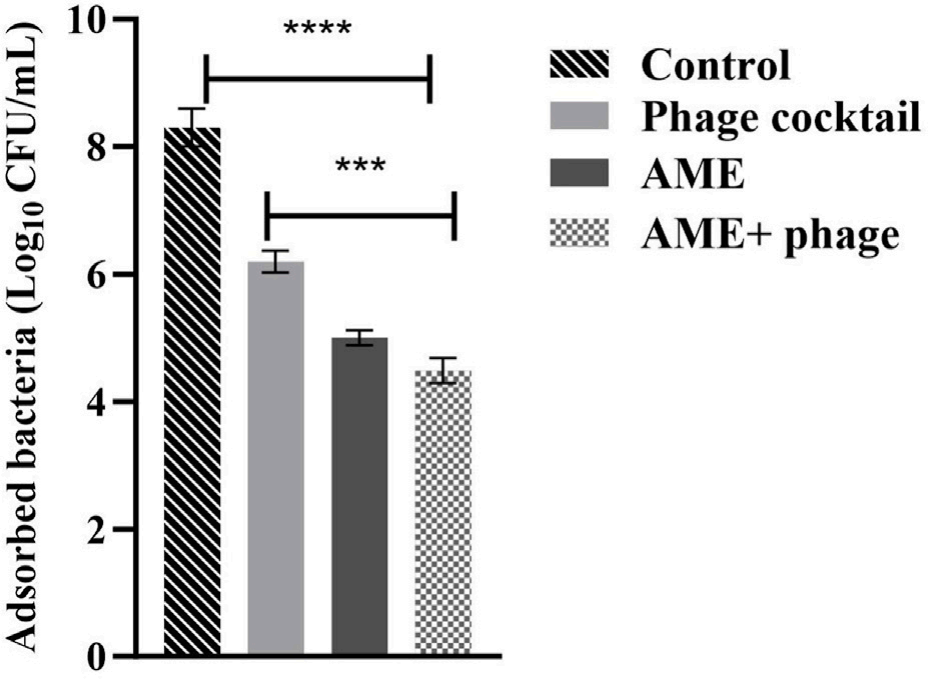
Effect of combined phage cocktail-extract on adhesion of *P. mirabilis* to a Vero cell line at optimum concentrations (MOI 1, and 125/mL, respectively). The statistical method used was the One-way ANOVA with post-hoc Tukey test. The stars represent as mentioned, ****: P< 0.0001, ***: P = 0.0002.

The adhesion assay on Vero cells showed that the combined treatment of phage cocktail (MOI 1) and the extract (125 μg/mL) decreased the adhesion of the bacteria to the Vero cells with 1.85 and 1.34 log as compared to the control and phage alone, respectively (P < 0.0001, *p* = 0.0002) (Figure 3).

### Phantom bladder model

**FIGURE 4:**
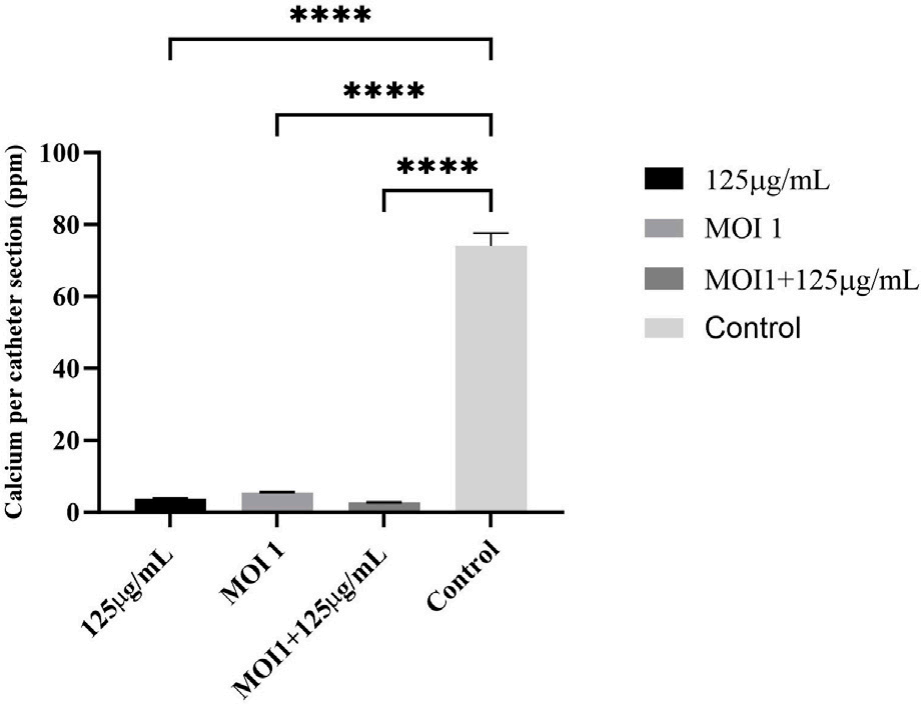
Calcium deposition on the catheter (ppm per section of catheter). Phage cocktail, AME, and combined extract-phage significantly reduced levels of encrustation as compared to the control. The statistical method used was the One-way ANOVA with Dunnett’s test and the P-value was calculated (P < 0.0001).

As demonstrated in Figure 4, the phage cocktail alone, AME alone, and the combined therapy all significantly reduced levels of encrustation as compared to the control. There was no difference in urine pH after treatment with the extract alone, or in combination with phage cocktail when compared to the control.

### Scanning electron microscopy for biofilm analysis

**FIGURE 5:**
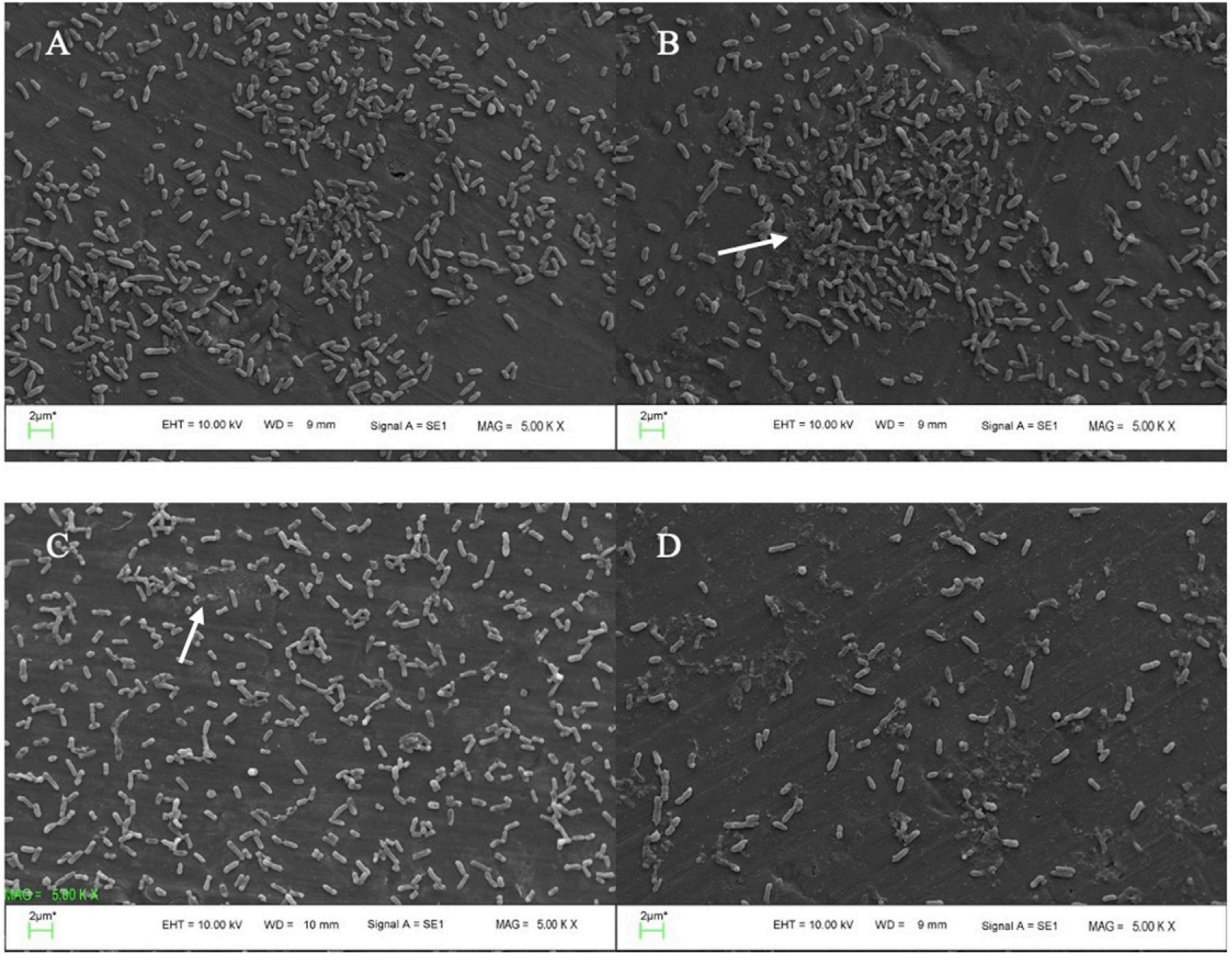
SEM micrographs of *P. mirabilis* biofilm. **(A)** biofilm of untreated *P. mirabilis* ATCC7002 control (without AME and Phage cocktail) showing bacterial cell condensation, and strong biofilm formation along with exopolysaccharide; **(B)** biofilm reduction using 125 μg/mL AME alone, in which the biofilm architecture displays scratches-like structures; The arrow indicates the disruption of the polysaccharide matrix of the biofilm. **(C)** phage cocktail treatment at MOI 1, led to changes in both bacterial morphology and the matrix structure; **(D)** combined phage cocktail (MOI 1) and AME (125 μg/mL) treatment showing efficient biofilm destruction and removal.

SEM micrograph revealed that the treatment led to changes in disruption of the polysaccharide matrix of biofilm and bacterial morphology (Figures 5 B, C) as compared to the untreated control (Figures 5A). Combined phage cocktail-AME treated biofilm induced more cell lysis and biofilm destruction compared to phage or AME alone treatment (Figures 5D).

### Real-time PCR of QS- and adhesion-related genes of combined therapy

**FIGURE 6:**
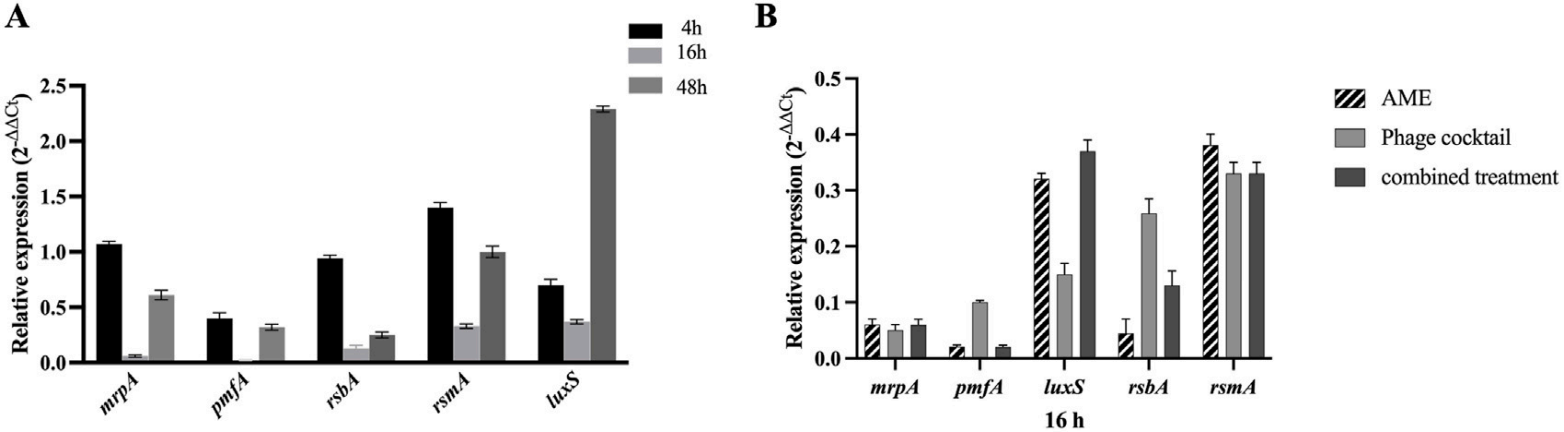
**(A)** Downregulation of *mrp*A, *pmf*A, *rsb*A, *rsm*A and *lux*S genes in *P. mirabilis* ATCC7002 in combined AME and Phage cocktail at optimal concentration and MOI (125 μg/mL, 1:1, respectively). The gene downregulation in combined treatment was more significant compared to individual treatment at 16-hour treatment (P < 0.0001). **(B)** Relative expression levels of the *mrp*A, *pmf*A, *rsb*A, *rsm*A, and *lux*S genes in various treatments with AME (125 μg/mL), phage cocktail (1:1,10^8^ PFU/mL), and their combination at optimal concentration and exposure time (16 h).

The effect of the combined phage cocktail - extract at optimum concentrations on the expression of QS- and adhesion-related genes, including *mrp*A, *pmf*A, *lux*S, *rsm*A, and *rsb*A, was evaluated for *P. mirabilis* ATCC after 4, 16, and 48 h. Expression of all examined genes was significantly downregulated after 16 h post-treatment with a combination of phage cocktail and AME ranging from 63% to 98% in comparison with the negative control (P< 0.0001) (Figure 6).

## Discussion

Bacteriophage therapy represents a promising novel alternative approach for the treatment of urinary tract infections (UTIs) (Breijyeh et al., 2020). However, one of the major concerns of phage therapy is the emergence of bacterial mutants that are resistant to phages (Torres-Barceló et al., 2022). This disadvantage has been compensated by different strategies, such as using phage cocktails (Haines et al., 2021; Kim et al., 2021; Hatfull et al., 2022) and combined treatment with phages and antibiotics (Malik et al., 2021). Medicinal plants and their compounds are being interestingly investigated for antimicrobial effects (Sher, 2009). Previous studies have shown the increased antibacterial activity of combined plant extracts with various bacterial pathogens such as *Streptococcus pyogenes*, *Staphylococcus aureus*, *Escherichia coli*, *Pseudomonas aeruginosa*, *Acinetobacter baumannii, Salmonella typhi* and *Klebsiella pneumoniae* (Manandhar et al., 2019; Danish et al., 2020; Mohanta et al., 2020). However, to date, very few studies have evaluated the antimicrobial activity of combined plant extract and phage cocktail against MDR bacteria. In this study, we assessed a strategy using the phage cocktail along with *A. maurorum* extract to control MDR *P. mirabilis* biofilm. Initially, the potential antiviral effect of the extract on the phage cocktail as antibacterial agents in the bacterial host was tested. The findings revealed that even higher concentrations of the AME exhibited no discernible impact on the titration and plaque-forming ability of the bacteriophage in the co-incubation assay, which makes the combined use of the compounds possible. Goldstein *et al.* observed a significant suppression of T2 bacteriophage replication in *E. coli* after combining with the cinnamon extract and cinnamaldehyde in a time exposure manner (Goldstein and Shumaker, 2019). In another study, Stachurska *et al.* reported that *Echinacea purpurea* (EP) and *Ruta graveolens* (RG) extracts at low concentrations show antiphage and bacterial stimulating activity. They proposed that the antagonism depends on the species of the phage and bacterial host (Stachurska et al., 2023).

In this study, we showed that the combination of AME and phage cocktails at optimal concentrations did not significantly reduce bacterial concentration when compared to the untreated or AME-treated control. However, the phage cocktail alone or in combination with the extract exhibits significant lytic activity against *P. mirabilis* isolates, which proposes that the phage cocktail drives the bacteriolytic activity. Our results agree with Pimchan et al.'s work on the plant extracts derived from *Stephania suberosa* roots, *Oroxylum indicum* fruits, and *Boesenbergia rotunda* rhizomes in combination with lytic phages against *E. coli*. They showed that the phages had no significant difference in antibacterial activity after 24 h; however, phages with extract significantly reduced the bacterial content in all cases for up to 6 h compared to the untreated and extract-only controls (Pimchan et al., 2018). Given the multifaceted nature of the interaction between the extract and phage on bacterial cells, several factors, such as the concentration and dosage of the extract or phage, as well as the duration of exposure, likely contribute to the observed variation in results. It is plausible that the interaction of the compounds within the extract and phage exerts influence over multiple aspects that merit consideration.

Moreover, the combined treatment’s anti-QS activity showed no difference compared to phage individual treatments. These results suggest that the presence of the phage cocktail likely did not exert an influence on QS and that the extract component primarily drove the anti-QS. Leon Flix et al. showed that using *P. aeruginosa* supplemented with penicillic acid as a QS inhibitor caused increasing productive infections of stationary-phase cells by two lytic phages (León-Félix and Villicaña, 2021). However, it is indisputable that QS exerts a significant influence on phage dynamics, impacting various aspects such as biofilm development, virulence, host colonization, and dispersal (Qin et al., 2017; Tan et al., 2020; Shah et al., 2021).

The effect of AME at a concentration of 125 μg/mL, when compared to a combination of phage and AME, did not exhibit a statistically significant impact on the biofilm inhibition assay conducted *in vitro* using microtiter plates with crystal violet staining. However, contrasting findings were observed in the phantom bladder model and scanning electron microscopy (SEM) analysis, where the combination treatment demonstrated a noteworthy decrease, highlighting the condition-dependent outcomes obtained from combination therapy in an *in vitro* and stimulating *in vivo* model. Our results showed that the interaction of bacteria, phage, and extract is more complex and multifaceted than what can be observed under controlled laboratory conditions. Several possible reasons include biological complexity, microenvironmental factors, pharmacokinetics and metabolism, immune system interactions, tissue complexity and interactions, and systemic effects (Niu et al., 2019; Podlacha et al., 2021). The substantial reduction in calcium deposition on the urinary catheter and the decreased bacterial attachment to the cell, achieved by optimizing the titer of AME and phage, potentially result from a synergistic interplay involving phage receptors. In combination therapy, the effect and interaction of each compound used must be assessed. This suggests that there may be an interaction between the quorum quenching activity of the crude extract (inhibition of bacterial communication) and the phage cocktail, leading to enhanced biofilm disruption. The genes under investigation in this study demonstrated a clear linkage to both biofilm formation and the QS phenomenon. The observed downregulation of these genes aligns with the decrease in both biofilm formation and QS activity, providing evidence for the significance of gene expression in these processes.

Overall, our study provides insights into the diverse mechanisms through which the combination of phytochemicals and bacteriophages can exert their effects on bacterial behavior and biofilm formation. By understanding these mechanisms, we can potentially develop novel strategies for combating bacterial infections and addressing the challenges posed by biofilms.

The synergistic utilization of phage therapy in combination with AME holds promising potential in the context of reducing biofilm formation. This combination approach demonstrates a favorable outlook for mitigating biofilm-related challenges, particularly by diminishing bacterial attachment to receptor sites and significantly reducing encrustation levels. Consequently, the application of this synergistic combination, specifically in the context of coated catheters or other medical instruments, could prove to be highly effective in combating biofilm-associated complications.

## Data Availability

The datasets presented in this study can be found in online repositories. The names of the repository/repositories and accession number(s) can be found in the article/Supplementary Material.

## Abbreviations

AMR: Antimicrobial resistance, AME: *Alhagi maurorum* extract, MDR: multi-drug resistant, SEM: Scanning electron microscopy, MOI: multiplicity of infection, MIC: minimum inhibitory concentration, ICU: intensive care units, PBS: phosphate-buffered saline, QS: quorum sensing, UTIs: urinary tract infections.
