# Supplementary figures and images for: Alhagi maurorum extract in combination with lytic phage cocktails: a promising therapeutic approach against biofilms of multi-drug resistant P. mirabilis
Source: Front Pharmacol. 2024 Dec 13;15:1483055. doi: 10.3389/fphar.2024.1483055 (PMC11671267; doi:10.3389/fphar.2024.1483055)

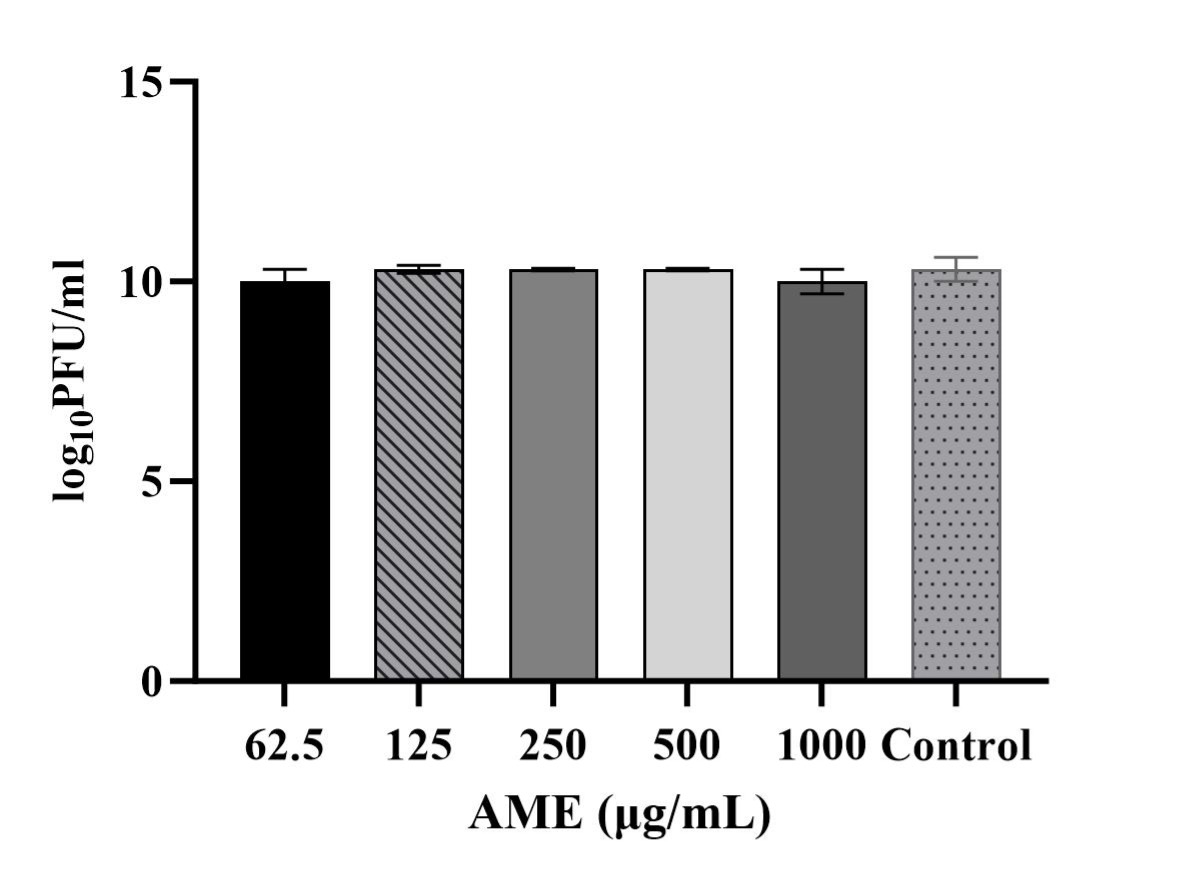

Supplement: Supplementary file 1 [file Image3.JPEG]

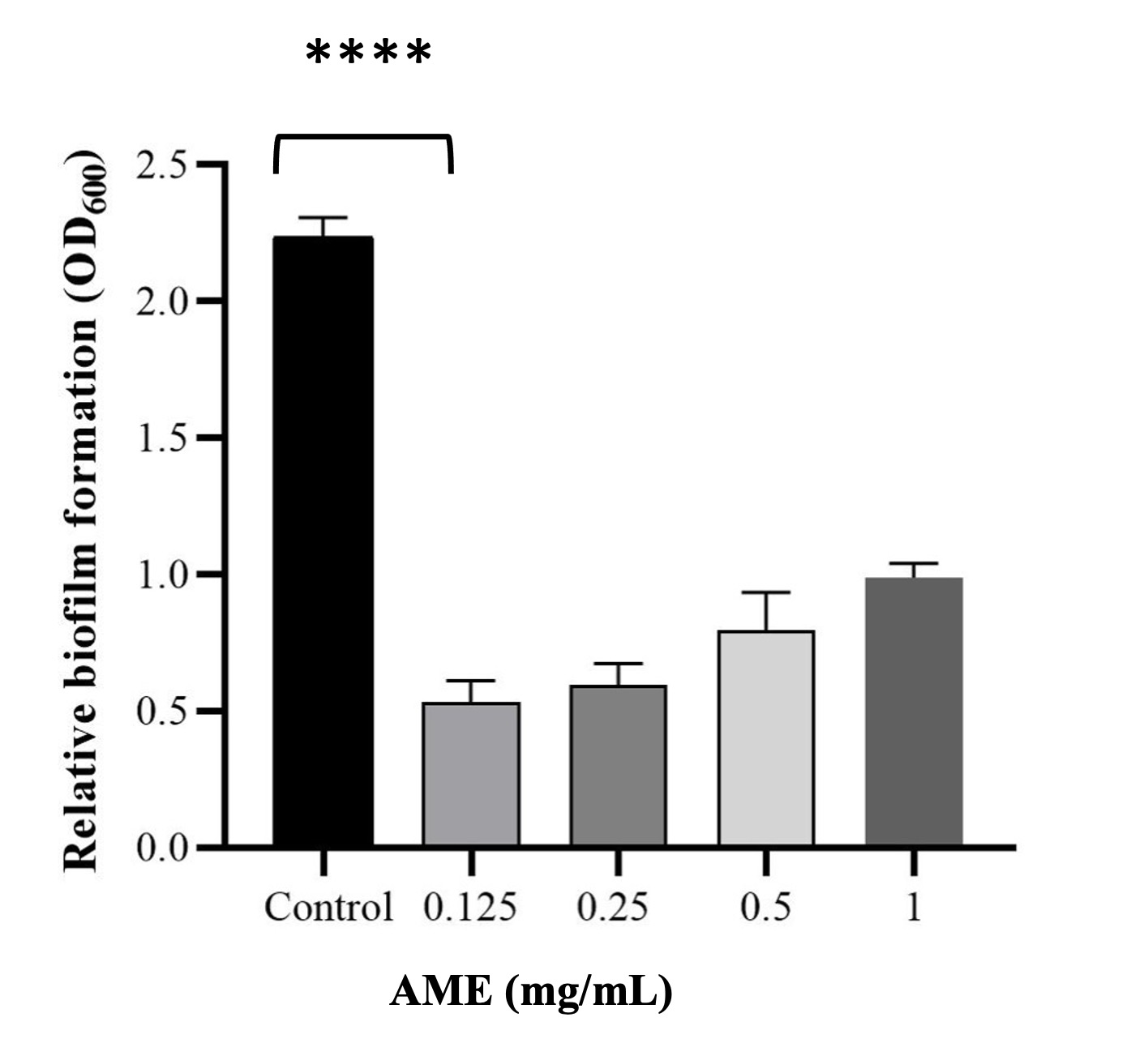

Supplement: Supplementary file 3 [file Image1.JPEG]

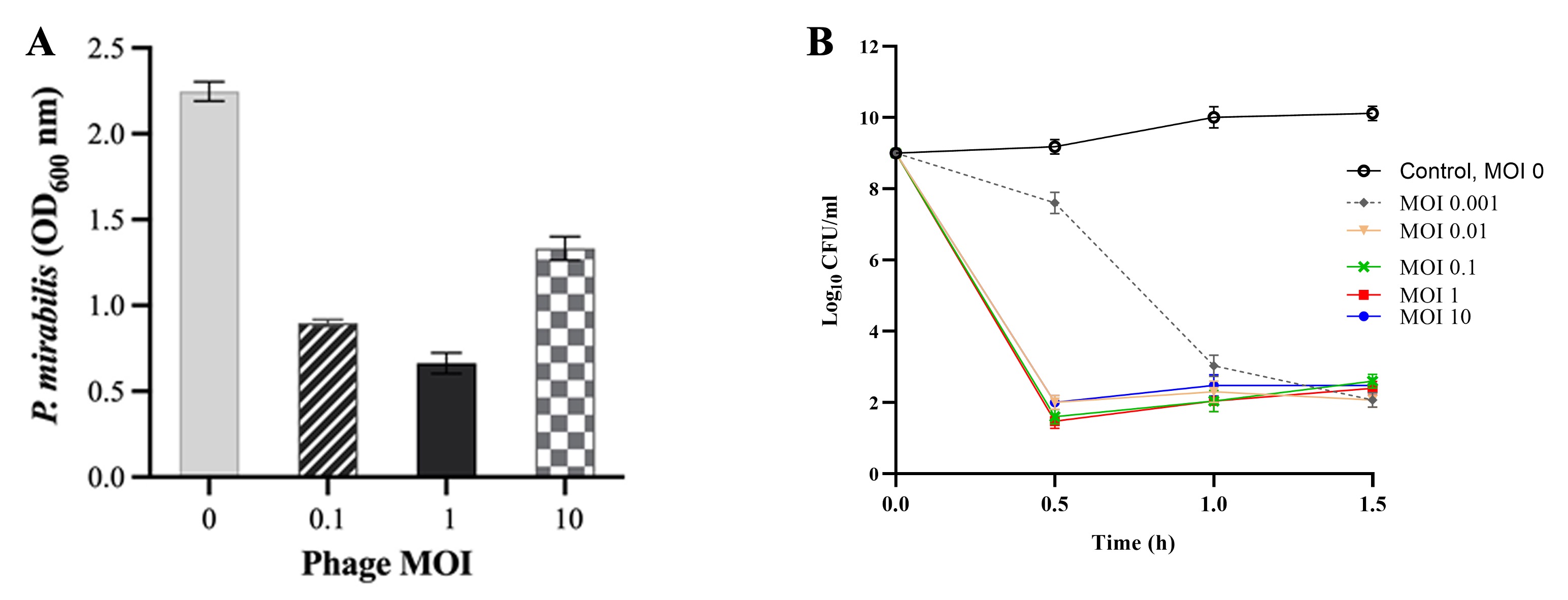

Supplement: Supplementary file 4 [file Image2.JPEG]
