# Supplementary material for: Alhagi maurorum extract in combination with lytic phage cocktails: a promising therapeutic approach against biofilms of multi-drug resistant P. mirabilis
Source: Front Pharmacol. 2024 Dec 13;15:1483055. doi: 10.3389/fphar.2024.1483055 (PMC11671267; doi:10.3389/fphar.2024.1483055)
Supplement: Supplementary file 2 [file Table1.DOCX]

| Isolate | Antibiotics | | | | | | | | | | | | | | | Biofilm formation Capacity/MDR |
| --- | --- | --- | --- | --- | --- | --- | --- | --- | --- | --- | --- | --- | --- | --- | --- | --- |
|  | AMC | SAM | ATM | TET | MEM | NOR_FX_ | CAZ | AM | OFX | CFM | SXT | CIP | NIT | AMK | CTX |  |
| Pm2 | R | R | R | R | R | R | S | R | R | S | R | R | R | S | R | Strong-MDR |
| Pm5 | S | S | R | R | R | S | S | S | R | S | R | R | R | S | S | Strong-MDR |
| Pm10 | S | S | S | R | S | R | R | R | R | S | R | R | R | S | S | Strong-MDR |
| Pm11 | S | S | R | R | R | R | S | S | R | S | R | R | R | S | S | Strong-MDR |
| Pm13 | S | S | R | R | S | S | S | S | R | S | R | R | R | S | S | Strong-MDR |
| Pm14 | S | S | S | R | S | S | R | S | S | R | S | S | R | S | R | Strong-MDR |
| Pm17 | S | S | S | R | R | S | S | R | S | R | S | R | R | S | R | Strong-MDR |
| Pm27 | S | S | S | R | S | R | S | S | R | S | R | R | R | S | S | Strong-MDR |
| Pm31 | R | S | S | R | R | R | S | R | R | R | R | R | R | R | S | Strong-MDR |
| Pm34 | R | S | R | R | R | R | R | R | R | R | R | R | R | R | R | Strong-MDR |
| Pm37 | R | S | R | R | R | R | R | R | R | R | R | R | R | R | R | Strong-MDR |
| Pm40 | S | S | S | R | S | R | S | R | R | S | R | R | R | S | S | Strong-MDR |

Supplementary Table1: Antimicrobial Profiles of selected isolates
